# Seed-specific expression of porcine verotoxigenic *Escherichia coli* antigens in tobacco plants as a potential model of edible vaccines

**DOI:** 10.1007/s11259-024-10318-y

**Published:** 2024-02-06

**Authors:** Serena Reggi, Matteo Dell’Anno, Antonella Baldi, Luciana Rossi

**Affiliations:** Department of Veterinary Medicine and Animal Sciences - DIVAS, University of Milan, Lodi, 26900 Italy

**Keywords:** Fimbriae, Shiga-like toxin, Post weaning diarrhoea, Engineered plants, *Nicotiana tabacum*, Alternatives to antibiotics

## Abstract

Vaccines can reduce the use of antibiotics by preventing specific infective diseases in pigs. Plant-based edible vaccines are particularly attractive because, upon oral ingestion via feed, they can elicit the local immune system against a foreign disease-causing organism. The aim of this study was to engineer two different independent lines of tobacco plants for the seed-specific expression of immunogenic proteins of VTEC as a model of an edible vaccine. For each antigen, fifty *Nicotiana tabacum* L. cv Xanthi leaf disks were transformed by agroinfection for the seed-specific expression of the structural parts of the fimbrial subunit FedF of F18 and the B-subunit of Vt2e genes. The synthetic genes, optimized by the codon adaptation index for their expression in tobacco, were inserted into expression cassettes under the control of β-conglycinin promoter. Regenerated tobacco plants (T0) were characterized by molecular and immunoenzymatic techniques. Our results showed that both FedF and Vt2eB genes were integrated into tobacco genome efficiently (> 80%) and they are also maintained in the second generation (T1). Western blotting analyses carried out on the positive producing lines, showed the tissue-specific expression in seeds and the temporal protein accumulation in the mid-late maturation phases. The enzyme-linked immunosorbent assay showed seed expression levels of 0.09 to 0.29% (from 138 to 444 µg/g of seeds) and 0.21 to 0.43% (from 321 to 658 µg/g of seeds) of total soluble protein for the FedF and Vt2eB antigens, respectively. This study confirmed the seed-specific expression of the selected antigens in plant seeds. The expression level is suitable for seed-based edible vaccination systems, which could represent a cost-effective way to prevent VTEC infection. Our findings encourage further in vivo studies focused on the activation of the local immune response.

**Supplementary Information:**

The online version contains supplementary material available at 10.1007/s11259-024-10318-y.

## Introduction

In pigs, pathogenic *Escherichia coli* can be classified into many pathotypes, based on virulence factors, bacterial adhesive properties to enterocytes, effects of adhesion on host cells, toxins production, and invasiveness (Luppi et al. 2016; Dubreuil et al. 2016). Verocytotoxin-producing *E. coli* (VTEC) strains previously named Shiga-like toxin *E. coli* (STEC), are major pathotypes often linked to piglets diseases such as enterotoxaemia, and oedema disease (OD) (Moxley 2022). VTEC strains, belonging to serogroups O138, O139, and O141, can produce verotoxin (Vt2e), which is responsible for the clinical signs and pathological lesions of OD (vascular damage, increases in vascular permeability, oedema in several tissues) (Ho et al. 2013). VTEC is characterized by two main significant virulence factors: verocytotoxin (Vt2e) and F18 fimbrial adhesin. Vt2e is a bipartite 70-kDa protein, composed of a single enzymatically active A subunit and five B subunits, that bind the toxin to the cell surface (Baldo et al. 2020).

F18 is a key fimbrial colonization factor of VTEC and is also involved in the development of the post-weaning disease (PWD) (Luise et al. 2019; García et al. 2020). The minor subunit FedF of F18 fimbriae is involved in the bacterial adhesion to intestinal brush borders and the development of local immunity (Tiels et al. 2008; Coddens et al. 2011; Moonens, 2012). The minor subunit FedF is a conserved region of F18 fimbria which binds to porcine epithelial cells (Smeds et al. 2003).

In pig livestock, PWD and OD lead to economic losses due to mortality, reduced piglet performances, and the cost of treatment, which includes fluid therapy and antimicrobials. However, the wide use of antibiotics in diarrhoea has led to the development of resistance in most of the gut-associated *E. coli* pathotypes of piglets and consequently therapeutic failures. Moreover, given the global emergence of antibiotic resistance, although it is not clear how antibiotic use in food-producing animals spreads resistant bacteria to humans, replacing antimicrobials is a key aim of EU policies (Tang et al. 2017). Alternatives to antibiotics that promote health status and prevent diseases are thus urgently needed (Coddens et al. 2017).

Oral vaccination represents an interesting strategy to control *E. coli* diseases, and a few studies have shown that it can protect against VTEC infections (Wen et al. 2006; Iannino et al. 2022). Orally delivered antigens have several advantages over other routes, including the induction of the mucosal immune response against infective agents that enter the body across the mucosal surface. Plant-based oral vaccines, currently developed for several human and animal diseases and investigated also in relation to COVID 19, offer a cost-effective, needleless, convenient, safe alternative to vaccine production (Sohrab 2020).

Seeds are an interesting target for heterologous antigenic protein expression due to the presence of natural protein storage organs. Plants can concentrate proteins in seeds, providing a stable environment for long-term storage (Scheller et al. 2006). Seed-based edible vaccines could help in the management of intensive rearing systems because the in-feed administration reduces labour costs and the stress related to the restraint of the animals (Rossi et al. 2014). Plant seeds, particularly tobacco seeds, are considered an optimal model for genetic engineering considering their suitability for producing high-value compounds without negative impacts on the soil, fertilization and environment, and for their potential scale-up to industrial production (Lau and Sun 2009; Nausch et al. 2016; Khan et al. 2019; Burnett and Burnett 2020). One of the limitations of plant seeds in general is content of secondary alkaloids that are not suitable for animal feeding. However, tobacco seeds do not accumulate these secondary metabolites such as nicotine, and tobacco seed cake is included in the EU catalogue of feed materials (Commission Regulation EU 2017/1017).

Previous studies by our research group showed that the oral administration of recombinant tobacco seeds expressing the major subunit of F18 fimbriae and the B subunit of Vt2e can induce a protective effect against VTEC challenge in piglet (Rossi et al. 2014, 2023). Despite the encouraging results, the adopted oral dose of 20 g of recombinant tobacco seeds, may be an obstacle for potential applicability in swine farming. In fact, the significant reduction in feed intake during the weaning transition of piglets can compromise intestinal health and the effectiveness of vaccination.

For these reasons, in the current study in order to reduce the amount of seeds for the immunization treatment, we considered the promoter of beta-conglycinin, an abundant storage protein in soybean seeds, in order to verify whether it triggers an enhancement in the expression level in seeds of heterologous proteins.

Given that Tiels et al. (2008) demonstrated that the FedF portion of F18 fimbriae possesses higher immunogenic potential compared to other fimbriae subunits, we focused on major virulence factors that can elicit an immune response and which, if lost or inactivated by specific antibodies, reduce *E. coli* pathogenicity.

The overall aim of the study was to engineer and characterize tobacco plants for the seed-specific expression of the B-subunit of Vt2e and the minor subunit FedF of F18 fimbriae as a potential model of edible vaccines against VTEC.

## Materials and methods

### Codon usage analysis and modification

The sequences of FedF (15–165 from mature peptide) and Vt2eB retrieved from gene sequences in the GenBank database (DQ914286 and AY437806, respectively) were analyzed by OptimumGene Algorithm (OptimumGene - Codon Optimization US Patent 8,326,547: Document Identifier US 20,110,081,708 A1). Synthetic genes studies and synthesis were performed by GenScript (Piscataway, NJ USA). The codon adaptation index (CAI) was upgraded from 0.76 to 0.94 for FedF and 0.95 for Vt2eB. GC adjusted content was equal to 38.70% for FedF, and 35.70% for Vt2eB. In addition, CpG dinucleotides content, mRNA secondary structure, cryptic splicing sites, premature polyA sites, internal chi sites and ribosomal binding sites, negative CpG islands, RNA instability motif (ARE), repeat sequences (direct repeat, reverse repeat, and Dyad repeat), and restriction sites that may interfere with cloning were considered for gene synthesis. To facilitate the direct subcloning in the expression vector, the *BamHI* and *SacI* sites of restriction were introduced at the extremity of the coding sequence (CDS).

### Construction of the binary plant expression vectors

The beta-conglycinin promoter gene (Acc.No.M13759, 1151 bp) was isolated from the leaves of soybean (*Glycine max* cv. Richland). The amplified gene region also included the sequences coding the signal peptides (62 aa) and the first codon of the mature proteins (V). To facilitate the subcloning of the promoter with the leader sequence in frame with the CDS, we inserted the restriction enzyme sites *XbaI* and *BamHI.* The sequences of oligonucleotides used for amplification are listed in Table 1 (congl-fw and congl-rv).

**Table 1:** Oligonucleotides pairs used for detection of FedF and Vt2eB genes, and β-conglycinin promoter

ID	Oligonucleotide sequence	Accession number	Amplicon size (bp)	Tm (°C)	PCR cycles
Congl-fw	**TCTAGA**GTTTTCAAATTTGAATTTTAATGTGTGTTG	M13759	1151	58	93°Cx5’ 93°Cx45’’ 55°Cx45’’ x30 72°Cx1’15’’ 72°Cx5’
Congl-rv	**GGATCC**CACCTTAAGGAGGTTGCAACGAGCGTGGCA			69	
Vt2eB-fv	**GGATCC**ATGAAGAAAATGTTCATCGCTG	X81417	276	60	95°Cx2’ 95°Cx 30’’ 55°Cx30’’ x35 72°Cx45’’ 72°Cx5’
Vt2eB-rv	**GAGCTC**TCAATTAAACTTAACCTGAGCAAAAC			60	
FedF-fw	**GGATCC**ATGAATTCTTCAGCTTCTTCAGC	DQ914286	471	61	95°Cx2’ 95°Cx30’’ 55°Cx30’’ x35 72°Cx45’’ 72°Cx5’
FedF-rv	**GAGCTC**TCATTTAGCAATAGCAGGAACATAG			60	

Restriction site of the expression vector used for cloning is presented in bold

**Fig. 1:**
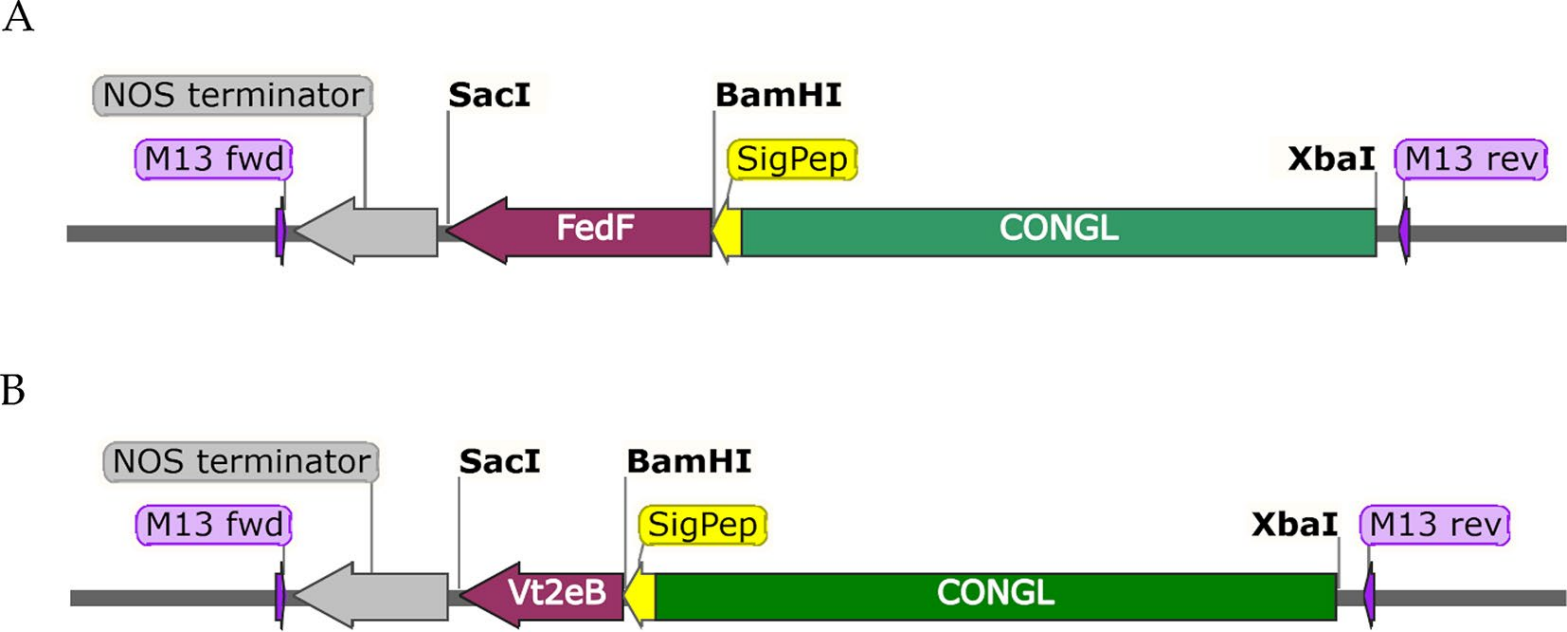
The expression cassettes. The FedF (15–165) and Vt2eB CDS were regulated by the conglycinin promoter and NOS terminator. **A**) The expression cassette from the binary vector pBI-Congl-FedF used for tobacco transformation. **B**) The expression cassette from the binary vector pBI-Congl-Vt2eB used for tobacco transformation

The amplified promoter was cloned in pGEMT-easy and sequenced to verify the correct amplification. The beta-conglycinin promoter sequence was inserted in *XbaI* -*SacI* sites into binary plant expression vector pBI101 (Clontech, Palo Alto CA, USA). The optimized genes were cloned in *BamHI*-*SacI*, in frame with signal peptide of the promoter. The expression cassette was completed with the addition of the Nos-terminator (Nos-T), which was cloned in the *SacI*-*EcoRI* site of pBI101 binary vector (Fig. 1A, B). The pBI101 vector included a selection marker (kanamycin resistance gene) to facilitate the screening of *E. coli* host and recombinant plants. We obtained the expression vectors named PBI-Congl-FedF and pBI-Congl-Vt2eB, respectively (Supplementary Fig. 1, Supplementary Fig. 2).

### Transformation of tobacco plants

Leaf discs of tobacco plants (*Nicotiana tabacum* L. cv Xanthi) were infected with *Agrobacterium tumefaciens* strain EHA105 which harbored the recombinant binary vectors by electroporation. Transfected callus tissues were selected by kanamycin resistance adding the antibiotic to the medium at a final concentration of 100 mg/L. Tobacco plant shoots were generated by the leaf disk infection method (Horsch et al. 1985). Only those that had rooted were screened for further analysis. Two different populations were produced (indicated as Vt2eB and FedF plants). Young leaves were collected and used for the next analysis. Regenerated plants (T0 plants) were transferred and grown in the greenhouse to produce seeds. Segregation for kanamycin resistance was observed in all independent transformed T0 tobacco lines. The seeds were germinated on MS medium with kanamycin (200 mg/L), and the ratio resistance/sensitive plants was observed. The classic Mendelian inheritance 3:1 (resistance/sensitive) and co-segregation with hygromycin confirmed the single insertion site. The kanamycin-positive plants produced T1 seeds.

*Nicotiana tabacum* L. cv Xanthi seeds were surface sterilized in 5% sodium hypochlorite for 5 min, and rinsed three times with sterile distilled water. They were then incubated in 70% ethanol for 1 min, and rinsed five times with sterile distilled water. Sterilized seeds were then germinated on MS medium containing sucrose 30 g/L, MS vitamin 1 mL/L, and Phytagel (Sigma-Aldrich, Saint Louis MO, USA) 2.5 g/L in Petri dishes. Seedlings were transferred to glass pots with the same culture medium for three weeks at 25 °C with a photoperiod of 12 h day/light. The regenerated T0 and T1 tobacco plants were grown in a biosafety greenhouse under controlled conditions (25–28 °C) and assessed for morphology and fertility.

The positive producing lines were selected and self-pollinated. The second generation was propagated in a greenhouse to produce the required amount of seeds.

### Molecular analysis

T0 or T1 generations were verified for the presence of the exogenous genes before flowering by PCR. Genomic DNA from young leaves of each plant was collected following Doyle and Doyle (1987). To screen for the presence of the transgene, the genomic DNA samples were used as the templates for PCR using the primers listed in Table 1 (Vt2eB-fw/Vt2eB-rv and FedF-fw/FedF-rv). Negative controls (DNA from untransformed tobacco plant leaves) were included with each experiment. Plants with positive PCR results were used for further studies.

T0 plants from FedF and T0 plants from Vt2eB were used in the Southern blot analysis to confirm the numbers of integrations of transgene in each plant. Young leaves (2 months) were harvested for genomic DNA extraction following Doyle and Doyle (1987). Briefly, DNA (10 µg) was subjected to digestion using XbaI, which is an enzyme that cleaves the expression vectors at a single site. The resulting DNA fragments were separated on a 0.8% (w/v) agarose gel, subsequently transferred onto a nylon membrane with a positive charge, and then subjected to hybridization with a probe (20 ng/ml) specific to the β-conglycinin promoter. This probe was labeled using the Dig High Prime DNA Labeling and Detection Kit II (Roche Diagnostics, Mannheim Germany), following the manufacturer’s instructions.

The positive control consisted of the vector (20 pg of pBI-Congl-Nos) restricted with the *EcoRV* enzyme. The detection was performed with the Dig High Prime DNA Labelling and Detection Kit II (Roche Diagnostics, Mannheim Germany) following the manufacturers’ instructions.

### Western blotting analysis

The T0 leaves, roots, culm, and seeds from both FedF and Vt2eB transformed plants (confirmed by PCR) were collected to verify their expression through Western blotting. Briefly, we extracted total soluble protein (TSP) from 100 mg of tobacco seeds and other anatomic sections of the plant (leaves, culm, and roots). This extraction was carried out using an extraction buffer consisting of 50 mM Tris-HCl (pH 8.0), 5 mM EDTA, 200 mM NaCl, 0.1% Triton X-100, and 1 mM PMSF in a 1:10 (w/v) ratio. The protein content was determined using Bradford reagent (Sigma Aldrich, St. Louis Missouri USA) using bovine serum albumin (BSA) as a standard. Approximately 50 µg of protein was mixed with SDS-loading buffer (containing β-mercaptoethanol), electrophoresis was performed in a 15% acrylamide gel, and transferred by electroblotting (using a solution of 25 mM Tris, 192 mM glycine, 20% methanol, at 30 V, overnight at 4 °C) onto a nitrocellulose membrane (Hybond ECL, GE Healthcare, Chicago Illinois USA). The membrane, which carried the bound protein, was submerged into 5% skimmed milk diluted in PBS-T solution, stirred for 60 min, washed, and subsequently incubated in the primary antibody solution. Lastly, the membrane was incubated with the corresponding secondary antibody peroxidase conjugate. Following several washes, the membrane was soaked in a chemiluminescent detection solution (ECL, GE Healthcare, Chicago Illinois USA). The Vt2e-B blot was probed with the antibody (1:500 v/v) against the Verotoxin II-B subunit (MYBioSource, San Diego CA USA) followed by hybridization with anti-mouse peroxidase conjugate (1:1000 v/v) (Sigma-Aldrich, Saint Louis, USA). The FedF blot was probed with the antibody (1:1000 v/v) against FedF (AbsoluteAntibody, Redcar, United Kingdom) followed by hybridization with anti-rabbit peroxidase conjugate, 1:2000 (Sigma-Aldrich, Saint Louis, USA). The Western blotting analysis was also used to confirm the seed-specific expression of the β-conglycinin promoter. The same quantity of protein extracted from seeds, leaf, culm, and root from FedF and Vt2e-B engineered plants were blotted and hybridized in the same conditions as specified above. To study the temporal expression of the β-conglycinin promoter, total proteins (30 µg) were isolated from FedF transgenic seeds at different ripening stages (indicated as stage I, II, III, IV, and V, collected every five days after flowering), loaded on 15% acrylamide gel in denaturing conditions. After blotting on nitrocellulose membrane, the hybridization with antiserum FedF was performed.

The sequence relative to FedF and Vt2e-B genes were analyzed by NetNGlyc 1.0 Server in order to determine possible glycosylation sites. The glycosylation site prediction analysis was performed on the primary structure according to Gupta and Brunak (2002) in order to estimate possible post-translational modifications of exogenous genes.

### Production of Vt2eB and FedF antigens in *E. coli*

The wild-type FedF and Vt2eB genes amplified from *E. coli* (from the private collection of the University of Milan, Italy) were cloned in pET28 vector (Novagen, USA). The positive clones (pET-FedF and pET-Vt2e-B) were transformed into a BL21 strain for induction by IPTG (isopropyl-β-D-1-thiogalactoside). The proteins were expressed and purified following the manufacturer’s instructions. A recombinant *E. coli* colony was grown overnight in LB (Luria and Bertani) medium supplemented with 30 µg/mL of kanamycin at 37 °C, stirred at 200 rpm. Subsequently, 1 mL of the culture was diluted in 100 mL of fresh LB medium (30 µg/mL kanamycin), and incubated. After reaching the optical density of 0.6 at 600 nm (OD_600_), the expression of recombinant proteins was induced by IPTG (1mM) and incubated for four hours. Bacterial cultures were centrifugated at 12 000 rpm for 15 min and microbial pellets were harvested. The soluble proteins and inclusion bodies were extracted following Sambrook and Russell (2001). The FedF and Vt2e-B protein induction were examined via 15% sodium dodecyl SDS-PAGE with Coomassie blue staining. The purified recombinant protein was quantified by Bradford reagent using BSA as the standard. The purified proteins were produced in the range of 1 mg/100 ml.

### RNA extraction, cDNA synthesis, and RT qPCR

RNA was extracted from tobacco seeds at different maturation stages (collected every five days from flowering). The RNA extraction was performed using the RNeasy Plant Mini Kit (Qiagen, Hilden Germany) with an additional DNase I (Thermo Fisher Scientific, Waltham, Massachusetts, USA) treatment. The RNA extract was verified by a spectrophotometer. The cDNA was obtained by the iScript cDNA synthesis kit (Bio-Rad, Hercules California USA), using a mix of random primers along with oligo (dT) and 2µg of total RNA. RT qPCR was performed using iTaq Universal SYBR GREEN Supermix (BIO-Rad, Hercules CA, USA) following the manufacturer’s instructions. The RTPCR was performed with the iQ5 system (Bio-Rad), with a setting block standard, an initial denaturation time of 95°C x 30 seconds, followed by 40 cycles at 95°C x 15 seconds and annealing/extension at 60°C x 30’’. Internal primers of FedF (Forward: ACTTTGACATGCCAGGCTGGAACTAC and Reverse: TGTACCGAATCCTACTTGTGACTGTTGC) and Vt2e-B Forward: CATCGCTGTTCTTTTCGCTTTGG and Reverse: TTGAGCTGATTGCAAAAGTGGTTGA) were designated to amplify a region of 150 and 172 bp, respectively. The expression level is expressed as 2^−ΔΔCT^ (Livak and Schmittgen 2001), using 18 S rRNA (Schmidt and Delaney 2010) as the housekeeping gene. The sample with the highest CT value, or the lowest observed gene expression, was used a calibrator.

### Quantitative ELISA

An immunoenzymatic reaction was set up to quantify the recombinant proteins expression levels. An indirect competitive enzyme-linked immunosorbent assay (IC-ELISA) was developed by coating recombinant mouse anti-Vt2eB (600 ng/mL, MYBioSource, San Diego CA USA) and rabbit anti-FedF (450 ng/mL, AbsoluteAntibody, Redcar, United Kingdom) antibodies onto a 96 microwell plate and incubated overnight at 4 °C. Plates were washed three times using 0.01 M PBS (pH 7) and subsequently blocked with 200 µL of 5% (w/v) BSA for 2 h at 37 °C. Mouse anti-Vt2eB and rabbit anti-FedF primary antibodies were diluted 1:200 and 1:300 respectively, and mixed with serial dilutions (1:1, 1:10 and 1:50) of tobacco seed total soluble protein extracted with the previously described procedure. From each sample, 100 µL were added to the coated wells of microplate and incubated at 37 °C for one hour. The plate was washed using PBST, and 100 µL secondary antibody (1:3000 diluted solution, Sigma-Aldrich, Saint Louis MO, USA) was added followed by one hour of incubation at 37 °C. Additional washing was performed with PBST, and 50 µL of TMB substrate were added in order to trigger the immunoenzymatic reaction. After 15 min of incubation at 37 °C the reaction was stopped by the addition of 150 µL of hydrochloric acid (HCl, 0.4 N), and absorbances were registered at 450 nm using a microplate reader (Bio-Rad, Hercules, CA, USA). The experiments were performed in triplicate. Standard curves for the quantification of antigens in tobacco seeds extracts were obtained by using recombinant Vt2e-B and FedF.

### Statistical analysis

The results of the gene expression were analyzed using JMP® Pro 15 (SAS Inst. Inc., Cary, NC, USA). All data were assessed for their normal distribution using the Shapiro-Wilk test. Relative expression values of RT-qPCR were analyzed using the one-way ANOVA. Multiple comparisons among groups were evaluated using Tukey’s Honest Significant Difference test (Tukey’s HSD). The results were presented as least squares means ± standard errors (SE). The means were considered different for *p* ≤ 0.05 and a trend towards significance when *p* ≤ 0.09.

## Results

### Tobacco transformation and regeneration

**Fig. 2:**
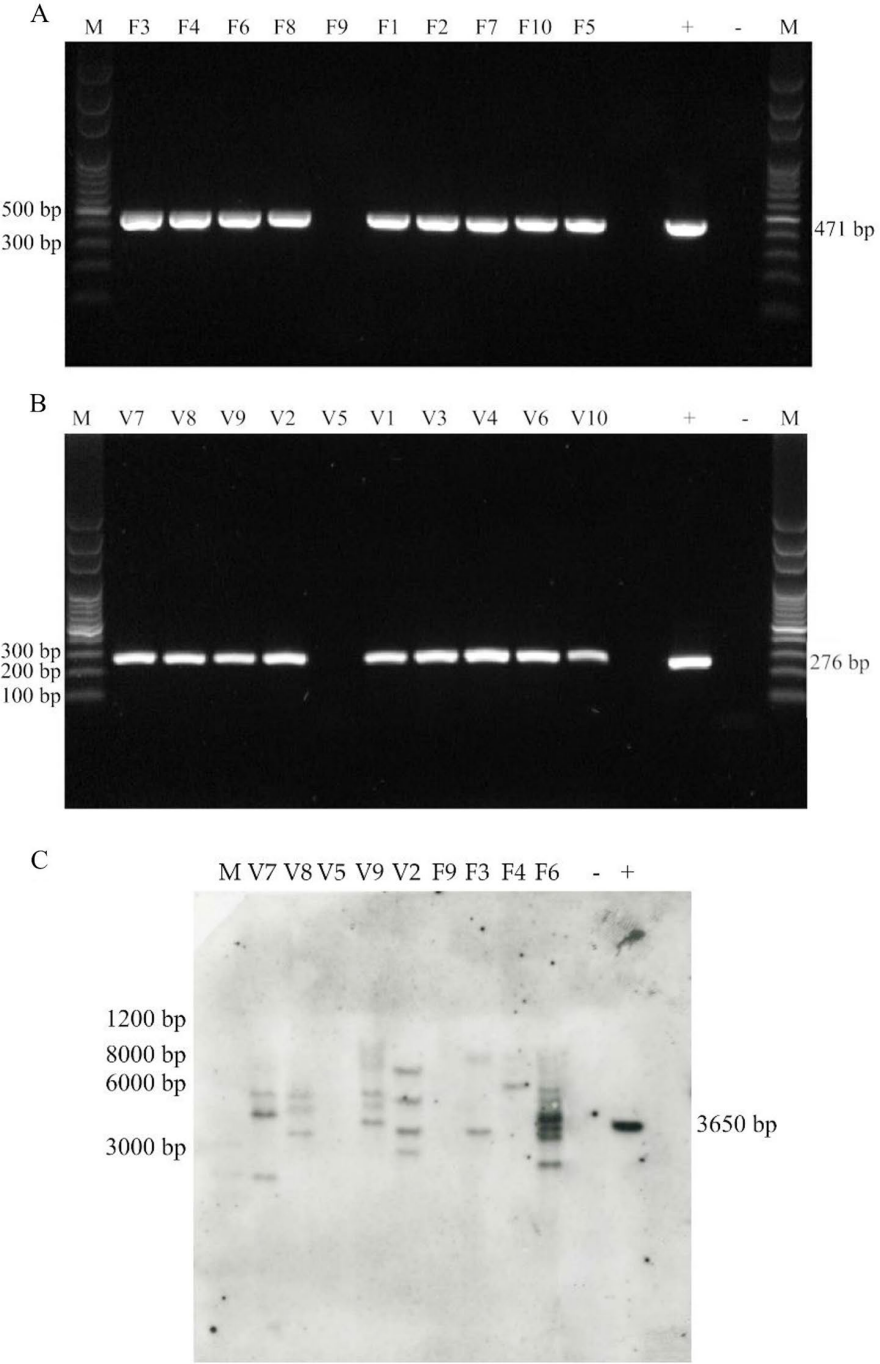
Agarose gel (1%) of PCR products on different T0 plants of *Nicotiana tabacum.***A**) Detection of FedF 15–165 gene in transgenic tobacco. Genomic DNA was amplified by specific primer (FedF-fw and FedF-rv) to detect a 471 fragment corresponding to the FedF gene (15–165 aa). F1-F10 selected transgenic plant Kanamycin resistance; M molecular weight marker; + positive control (pBI-congl-F18); - negative control (untransformed tobacco). **B**) detection of Vt2eB gene in transgenic tobacco. Genomic DNA was amplified by specific primer (Vt2e-fw and Vt2e-rv) to detect a 276 fragment corresponding to the Vt2eB gene. V1-V10 selected transgenic plant Kanamycin resistance; M molecular weight marker; + positive control (pBI-congl-F18); - negative control (untransformed tobacco). **C)** Southern Blot. M molecular weight marker, V7-V9 tobacco plants Vt2eB (V5, V8, V9, and V2 plants PCR positive, V5 plant PCR negative); F3-F9 tobacco plants FedF (F3, F4, and F6 plant PCR positive, F9 plant PCR negative); – untransformed tobacco plant (negative control); + positive control

The engineered vectors were used to generate, by agroinfection, lines of recombinant plants, expressing FedF and Vt2e-B antigens, respectively. About 50 leaf disks were infected for each transformation, leading to at least 20 kanamycin-resistant plants. The presence of the exogenous genes was checked by PCR as the first screening on T_0_ generation plants using the gene-specific primers listed in Table 1. A total of 81% of FedF plants were identified by the presence of a 471 bp fragment (Fig. 2A), corresponding at the same size observed in the positive control. A total of 84% of Vt2e-B plants were identified by the presence of a 276 bp fragment (Fig. 2B), corresponding to amplified observed in positive control. T0 transformed plants were obtained and grown under greenhouse conditions, and assessed for morphology and fertility. The engineered FedF and Vt2e-B plants were phenotypically and morphologically indistinguishable from wild-type lines. At the same time, the T0 seed was checked for Kanamycin segregation. The time of germination for both transformations showed similar results compared to a previous study conducted by our research group (from 5 to 7 days) (Onelli et al. 2017).

Some T0 plants were analyzed by Southern blotting. All FedF and Vt2e-B, PCR-positive, engineered plants showed positive hybridization, and most of the hybridization bands were between 2.5 and 10 kb. The FedF and Vt2e-B, PCR-negative, plants were confirmed to be negative. No hybridization bands were found in the untransformed tobacco plants used as a control. The engineered plants carried more copies of the exogenous genes FedF and Vt2eB. All T0 plants analyzed, revealed unique and complex hybridization patterns, indicating that these plants had indeed originated from independents events (Fig. 2C). Southern blot analysis confirmed that exogenous genes were integrated into the tobacco genome. The segregation for kanamycin resistance of plant checked by Southern blotting showed that plants with multiple copies of transgenes did not follow the classic Mendelian inheritance 3:1 (resistant to sensitive), thus confirming multiple insertion sites.

### Expression of Vt2e-B and FedF

**Fig. 3:**
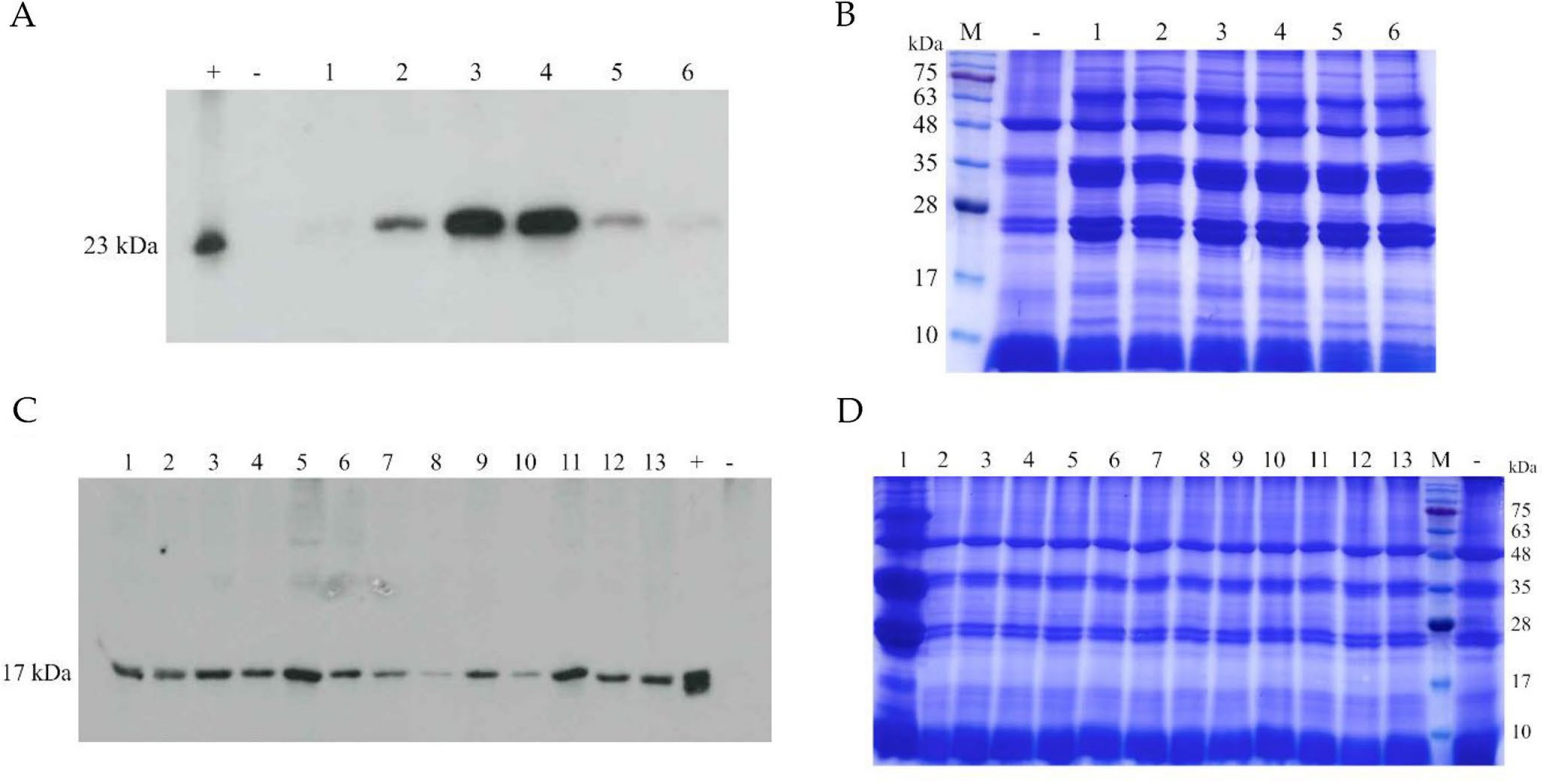
Western blot of T0 transformed tobacco seeds. **A**) 1–6 different FedF tobacco seeds (loaded 50 µg of TSP), + positive control (rFedF from *E. coli*) (50 ng); - negative control (untransformed tobacco). **B**) SDS-page Coomassie stained of 1–6 FedF tobacco seed extracts (loaded 50 µg of TSP), M: protein ladder 10–180 kDa (BlueStar Prestained Protein Marker, Nippon Genetics, Düren, Germany), - negative control (untransformed tobacco). **C**) 1–13 Vt2eB tobacco seeds (loaded 50 µg of TSP); + 100ng of rVt2eB; - untransformed tobacco seeds. **D**) SDS-page Coomassie stained of 1–13 Vt2eB tobacco seed extracts (loaded 50 µg of TSP), M: protein ladder 10–180 kDa (BlueStar Prestained Protein Marker, Nippon Genetics, Düren, Germany), - negative control (untransformed tobacco)

All the T0 plants, which were PCR positive, were analyzed by western blotting. The 65% of FedF plants showed the presence of a protein with a molecular weight of about 23 kDa, similar to the positive control (FedF recombinant produced in BL21 *E. coli*). No signal was detected in untransformed tobacco plants, thus confirming the specificity of the signal (Fig. 3A).

A total of 71% of Vt2e-B plants showed the presence of a protein of a molecular mass of about 17 KDa, similar to the positive control (Vt2eB recombinant produced in BL21 *E. coli*; Fig. 3C). No signal was detected in WT tobacco plants, again confirming the specificity of the signal.

The FedF and Vt2eB expression levels analyzed by ELISA assays showed that FedF was accumulated in the seeds in a variable range from 0.09 to 0.29% of the total soluble protein (TSP), corresponding to 138–444 µg/g of seeds; and Vt2eB from 0.21 to 0.43% of TSP, corresponding to 321–658 µg/g of seeds. Wild type tobacco seeds did not show any interference in the quantification of recombinant proteins registering values < 1 ng/mL in TSP extracted from 100 mg of tobacco seeds.

**Fig. 4:**
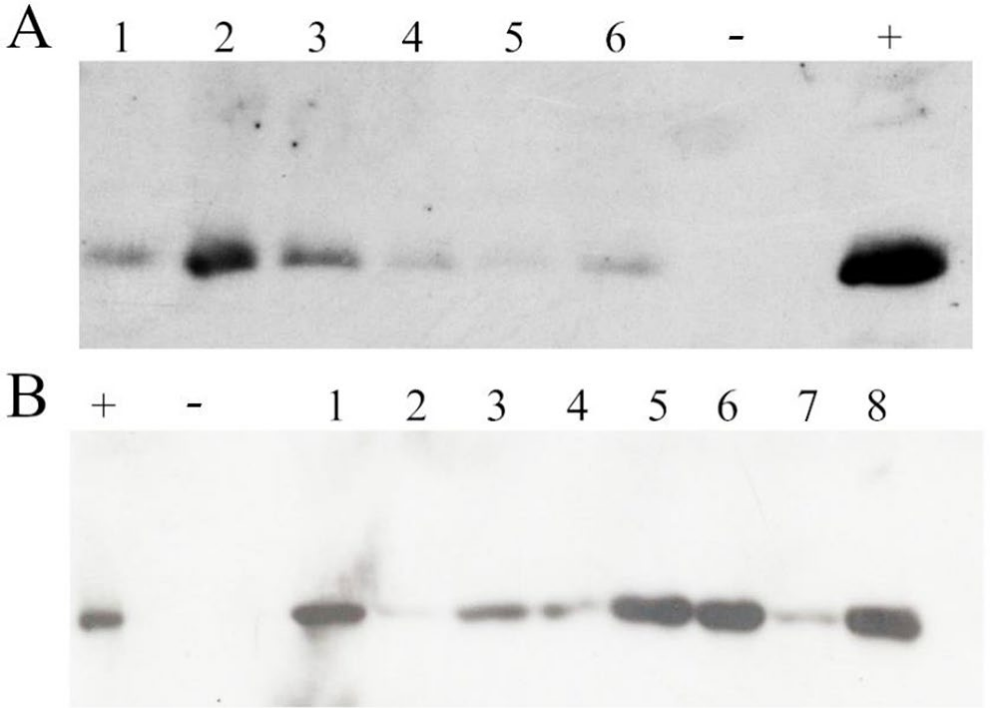
Western blotting of T1 plants. **A**) 1–3 different T1 seeds derived from T0 FedF plant line No. 2 (F2); 4–6 different T1 seeds derived from T0 FedF plant line No. 5 (F5); - negative control (untransformed tobacco); + rFedF from *E. coli* (100 ng). **B**) + rVt2eB from *E. coli* (100ng); - negative control (untransformed tobacco); 1–4 different T1 seeds derived from T0 Vt2eB plant line No. 2 (V2); 5–8 different T1 seeds derived from T0 Vt2eB plant line No. 7 (V7)

At least three T1 tobacco plants Kanamycin-resistant were randomly selected from each different T0 line. The presence of FedF and Vt2eB transgene was first confirmed by PCR, and the protein expression was checked by Western blotting (Fig. 4A and B). FedF and Vt2eB proteins were expressed in the next generation T1, confirming that there had been no silencing.

Glycosylation prediction *in silico* analyses of FedF and Vt2eB sequences by NetNGlyc 1.0 Server showed that FedF presents one potential site of glycosylation in position 21 (NTT), and that Vt2eB did not present any possible glycosylation site. This was confirmed by Western blotting, where the positive signal for both proteins was similar to the control positive expressed in *E. coli* BL21.

### Seed-specific and temporal expression of the conglycinin promoter

**Fig. 5:**
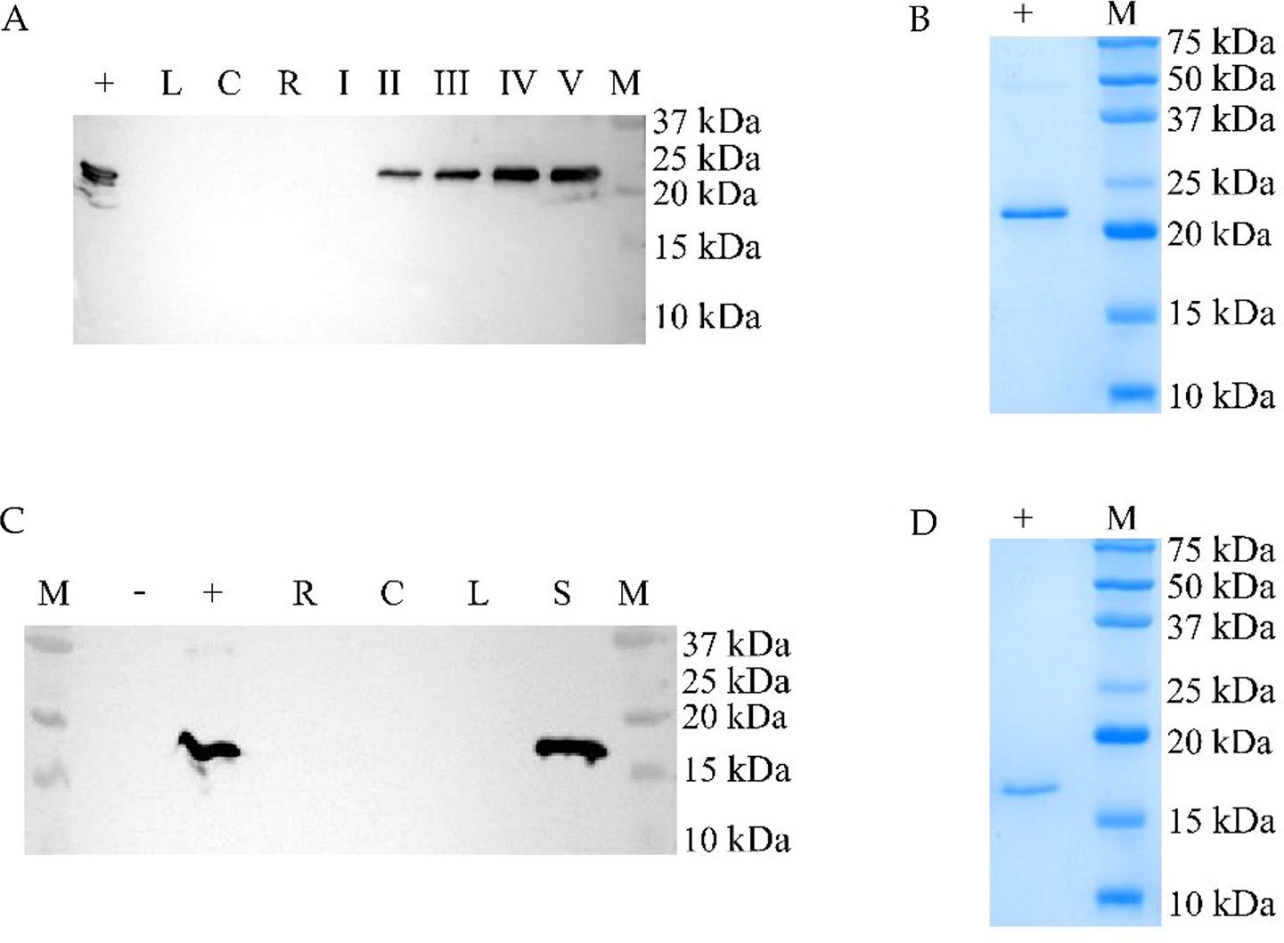
Seed-expression of FedF and Vt2eB. **A**) Seed-specific expression of FedF tobacco protein. + positive control rFedF from *E. coli*; L leaf proteins from FedF tobacco plant; C culm proteins from FedF tobacco plant; R root proteins from FedF tobacco plant. I-II-III-IV-V seed proteins at different stages of ripening from FedF tobacco plants. I: 5, II: 10, III: 15, IV: 20, V: 25 days after flowering; M molecular weight marker (Precision Plus Protein Marker, Bio-Rad, Hercules, CA, USA). **B**) SDS-PAGE 15% acrylamide gel Coomassie stained of FedF positive control (+) and molecular weight marker (M; Precision Plus Protein Marker, Bio-Rad, Hercules, CA, USA). **C**) Seed-specific expression of Vt2eB tobacco protein. M molecular weight marker (Precision Plus Protein Marker, Bio-Rad, Hercules, CA, USA); - negative control (untransformed tobacco); R root proteins from Vt2eB tobacco plant; C culm proteins from Vt2eB tobacco plant; L leaf proteins from Vt2eB tobacco plant. S seed proteins from Vt2eB tobacco plant. **D**) SDS-PAGE 15% acrylamide gel Coomassie stained of Vt2eB positive control (+) and molecular weight marker (M; Precision Plus Protein Marker, Bio-Rad, Hercules, CA, USA).

The specificity of the soybean promoter conglycinin was verified in the tobacco heterologous system as being tobacco. As shown in Fig. 5A and B, the signal FedF and Vt2eB proteins were detected only in the seeds, while the leaf, culm, and roots showed an absence of positive hybridization after a prolonged exposure. FedF seeds at different stages of ripening were collected and analyzed by Western blotting. As shown in Fig. 5A, FedF protein becomes detected later, from 10 days after flowering in mid maturation (stage II), and rapidly reached the plateau and then remained at the maximum level during the following ripening stages.

**Fig. 6:**
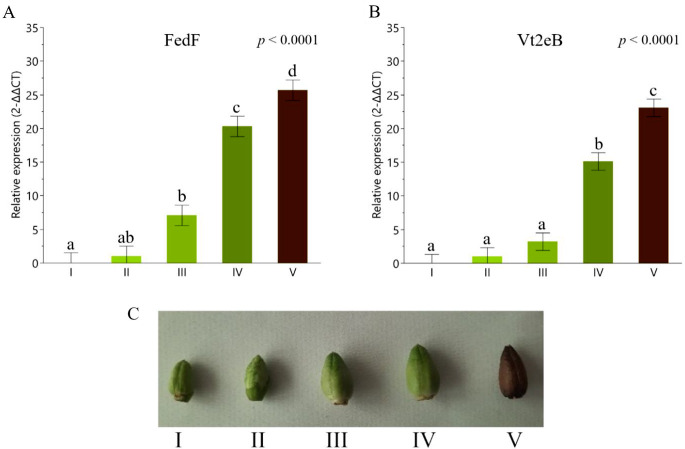
Relative expression of engineered plants. **A**) Seed-specific relative expression of FedF tobacco protein analyzed by RT-qPCR. **B**) Seed-specific relative expression of Vt2eB tobacco protein analyzed by RT qPCR. **C)** Tobacco seed capsules harvested at different stages of ripening. I: 5, II: 10, III: 15, IV: 20, V: 25 days after flowering. Data are expressed as least squares means ± standard error. Different lowercase letters indicate statistically significant differences (*p* < 0.0001)

This result was confirmed by RT-qPCR for both genes. As shown in Fig. 6A and B, FedF and Vt2eB proteins start accumulating at stage II (10 DAF) and accumulate rapidly at stage III (15 DAF; Fig. 6C). A higher relative expression of antigens was observed from stages III to V and from IV to V, for FedF and Vt2eB, respectively (*p* < 0.0001). The highest values of relative gene expression were found for stage V for both FedF and Vt2eB plants (*p* < 0.0001).

## Discussion

Engineered plants could be used as platform for antigen production and the final delivery systems of edible vaccines (Sahai et al. 2013). Plant-based immunogenic antigen production systems, such as transgenic or xenogenic plants that contain agents that trigger an animal’s immune response, are an interesting solution for the prevention and control of various diseases in human and veterinary medicine (Daniell et al. 2009; Kurup and Thomas 2020). Edible vaccines could potentially reduce the use of antibiotics in the veterinary field. In this study, we focused on main VTEC *E. coli* pathotypes of the swine species. The general idea was to produce antibodies against fimbriae and verotoxin, using tobacco engineered seeds for oral vaccination, aimed at reducing the intestinal colonization of pathogenic *E. coli* F18+ (Hur and Lee 2012). As antigens we used the immunogenic portions of virulence factors represented by the non-toxic part of the verocytotoxin type 2 (B subunit) and a fimbrial protein (FedF). Vt2eB has no enzymatic activity and is recognized as an immunogenic protein with adjuvant properties. Fimbriae are also thought to be very good immunogen particles because they are proteinaceous and contain a set of highly repetitive epitopes. The portion 15–165 of FedF is a stable domain, natural glycan receptor. Moones et al. (2012) confirmed that the N-terminal domain of FedF (15–165) determines F18-mediated adhesion to pig enterocytes.

We obtained different lines of transgenic tobacco plants expressing FedF and Vt2eB. Over 80% of the plants regenerated were positive to PCR for FedF and Vt2eB. The Southern blotting analysis revealed that most of the primary transformants contained several copies of the transgene, as typically reported in transformed plants via *Agrobacterium* species. These results indicate that a dissimilar pattern of hybridization products was obtained from different plants, suggesting different gene copy numbers and integration of the transgene into sites with variable transcription competencies. Transgenic single copy events are desirable because they follow a Mendelian segregation pattern and thus a lower possibility of gene silencing (Koprek et al. 2001; De Paepe et al. 2012). We analyzed the T1 populations, Western blotting confirmed the the FedF and Vt2eB protein were expressed in the next generation. The observed variability in the expression levels is typical of the primary transgenic lines (Kohli et al. 2006). This variability can be explained by the transgene copy number. More copies do not directly result in a higher protein expression level, however this variability can be explained by positional insertion in the tobacco genome. We also demonstrated that the transformed lines of *Nicotiana tabacum* express FedF and VT2eB proteins specifically in seeds. The seed expression was efficiently obtained using the β-conglycinin promoter. β-conglycinin is a storage protein that accumulates high levels only in soybean seeds. According to data reported from the analysis of promoters used for the expression of recombinant proteins in seeds of tobacco plants (Chamberland et al. 1992), the expression of FedF and Vt2eB proteins in transgenic tobacco plants is specific for seed tissues. Immunoblotting performed on leaves, roots, and culm protein extracts of positive plants did not reveal the presence of recombinant proteins. According to Chen et al. (1989), the proteins under β-conglycinin promoter were accumulated only in transgenic seeds during the mid-to-late stages of seed development as in soybean plants. Compared to previous studies, the use of β-conglycinin promoter helped to ensure a seed-specific production comparable to results achieved with a globulin promoter (Rossi et al. 2013a).

In a previous work (Rossi et al. 2023) we demonstrated that oral administration of tobacco seeds producing Vt2eB and FedA proteins in mice, after a challenge with O138 *E. coli* strain, had a protective effect and improved the animal performance. The present study represents a subsequent step, broadening the vaccination coverage aimed at an experimental antigen production system for future application as a plant-based vaccine against the most important VTEC strains. Information on codon usage BIAS has already been used to modify genes for improved expression in heterologous systems, such as tobacco.

The maximization of the expression in seeds obtained by CAI and GC% optimization supported the correct quantity of antigens for oral administration that would enable fewer seeds to be used in order to prevent any negative effects on feed palatability and the nutrient composition of the animal diet. RT-qPCR revealed that exogenous genes reached their maximum level of mRNA transcription after 25 days from the flowering stage. IC-ELISA enabled the quantification of recombinant proteins also in a complex matrix of tobacco seeds, in line with a previous study that adopted indirect ELISA for the evaluation of the expression levels in transgenic plant seeds (Reggi et al. 2023). Compared to the previous studies published by our research group, the quantity of expressed Vt2eB protein was approximately 1.5 times higher (0.3% of TSP vs. 0.43% of TSP) (Rossi et al. 2013a), and FedF was detected at a similar level of FedA expressed in transgenic tobacco seeds (Rossi et al. 2014). Although FedF and FedA could not be directly compared in terms of immunogenic potential due to their structural differences, FedF has been proposed as a preferable antigen considering its immunogenic potential and the tobacco-seed expression (Tiels et al. 2007; Rossi et al. 2013a). The obtained data could be interesting in decreasing the amount of seeds needed for piglet immunization. Tiels et al. (2008) provided 3 mg of F18 fimbria for oral immunization in piglets, we assume to reach the same amount of antigen by feeding 5 g of seeds from FedF line and 2.5 of seeds from the Vt2eB line of the highest expression engineered tobacco plants with a single administration. In our previous paper (Rossi et al. 2014), 10 g of FedA seeds and 10 g of Vt2eB seeds were used for oral delivery. In the current study, the developed multivalent oral vaccination platform represents an improvement considering the limitation of the gastric volume of piglets, the average daily feed intake at weaning and the balance of nutritional requirements of the diet.

The expression levels of 0.1-1% of TSP in FedF and Vt2eB xenogenic seeds have been considered competitive for antigen production by Twyman et al. (2003), which underlined that this range of recombinant is necessary to propose the plant as practical system compared to other expression technologies (Twyman et al. 2003).

Using of tobacco seeds for the delivery of antigenic proteins means that there is less chance of accidental entry of pharmaceuticals into the human food chain. Tobacco seeds are not used in human nutrition and tobacco seed cake, and despite being included in the catalog of Feed Materials (ID: 000752-EN, Reg. EU 2017/1017), they are not widely used. The inclusion of tobacco seeds in the piglet diet covered the nutritional requirements without affecting growth, feed efficiency, and metabolic parameters, and can thus be used as a protein source (Rossi et al. 2013a, b). The production of antigens in seeds has a long storage period and a natural encapsulation in the tissues of the expression host (Chan and Daniell 2015). This encapsulation enables the antigen to be protected against rapid and complete degradation, and to be gradually released as host tissues are digested (Rossi et al. 2013b). Each tobacco capsule contains on average 200 mg of seeds and each inflorescence possesses more than 60 capsules. A single tobacco plant produces and average of 12–16 g of seeds in a greenhouse culture. Considering an immunization protocol with four administrations, approximately two tobacco plants would provide 5 g/dose of FedF seeds and about one tobacco plant of Vt2eB would provide 2.5 g/dose of Vt2eB for each piglet. On a large scale, considering a density of 30,000 plants/ha the production yields could reach 360–480 kg/seeds/ha.

Pollen dispersal is under debate in terms of limiting the spread of exogenous genes. Transgene flows could be prevented through integrated confinement systems using various strategies. For example, the confinement level 1 uses guard rows (pollen-trap rows) that attract pollinating insects to prevent pollen-mediated gene flow from the engineered plants (Halsey 2006). Although, country in the EU has a specific regulation on GMO cultivation (Directive EC 2001/18), plant-based vaccine systems are included in the field of medical molecular farming, which is specific sector of GMO production that adopts the vertical farming production systems under high standard culturing conditions for manufacturing biopharmaceuticals (Buyel 2019; Lico et al. 2020; Huebbers and Buyel 2021).

Since a large number of pathogens can find access through mucosal surfaces in respiratory, gastrointestinal and urogenital tracts as their pivotal routes of entry into the host, the foremost line of defense is thus the mucosal immunity. The most efficient path of mucosa immunization is the oral route due to the ability of edible vaccines to stimulate antibody mediated and cell mediated immune responses. Therefore, engineered plants expressing antigens could potentially release epitopes into the gut environment. M cells placed on Payer’s patches and gut-associated lymphoid tissue, could transfer immunogenic proteins to macrophages and lymphocytes, thus producing IgG, IgE and local IgA response and memory cells, which counterbalance the attack by real infective agents (Su et al. 2016).

Future studies could evaluate whether the feed administration of the combination of the two lines of tobacco seeds, engineered for the expression of the main virulence factors of *E. coli* pathotypes is protective against the wild type *E. coli* infection in weaned piglets.

### Electronic supplementary material

Below is the link to the electronic supplementary material.

Supplementary Material 1

## Data Availability

No datasets were generated or analysed during the current study.

## Declarations

### Patents

The innovation of this study is under patent n° 102021000006461 (18/03/2021, Italy).
