# Supplementary material for: Seed-specific expression of porcine verotoxigenic Escherichia coli antigens in tobacco plants as a potential model of edible vaccines
Source: Vet Res Commun. 2024 Feb 6;48(3):1435–47. doi: 10.1007/s11259-024-10318-y (PMC11147939; doi:10.1007/s11259-024-10318-y)
Supplement: Supplementary file 1 — Supplementary Material 1 [file 11259_2024_10318_MOESM1_ESM.docx]

**Supplementary Materials**


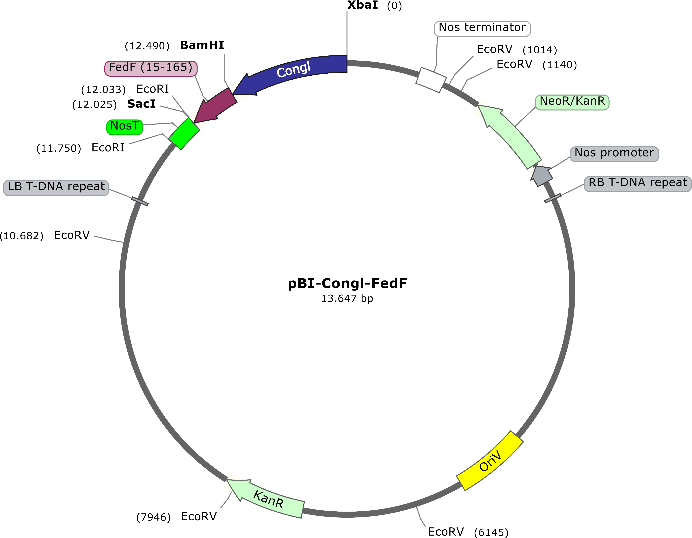


**Supplementary Figure 1.** Binary vector pBI-Congl-FedF used for tobacco transformation.


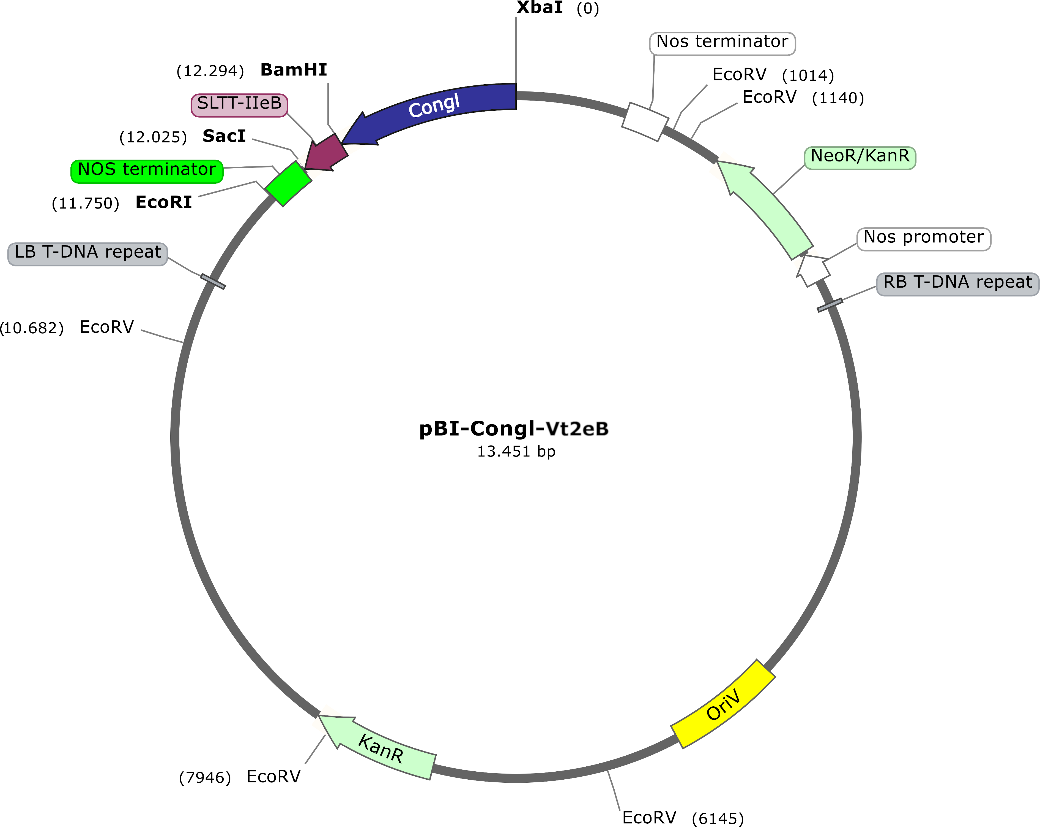


**Supplementary Figure 2.** Binary vector pBI-Congl-Vt2eB used for tobacco transformation.
